# Model errors in tree biomass estimates computed with an approximation to a missing covariance matrix

**DOI:** 10.1186/s13021-015-0031-8

**Published:** 2015-09-17

**Authors:** Steen Magnussen, Oswaldo Ismael Carillo Negrete

**Affiliations:** 1 Natural Resources Canada, 506 West Burnside Road, Victoria, BC V8Z 1M5 Canada; 2 CONAFOR, Periférico Poniente #5360 Col. San Juan de Ocotán, C.P. 45019 Zapopan, Jalisco Mexico

**Keywords:** Linear regression, Nonlinear regression, Weighted regression, Residual variance, Robust estimation, Parametric bootstrap

## Abstract

**Background:**

Biomass and carbon estimation has become a priority in national and regional forest inventories. Biomass of individual trees is estimated using biomass equations. A covariance matrix for the parameters in a biomass equation is needed for the computation of an estimate of the model error in a tree level estimate of biomass. Unfortunately, many biomass equations do not provide key statistics for a direct estimation of model errors. This study proposes three new procedures for recovering missing statistics from available estimates of a coefficient of determination and sample size. They are complementary to a recently published study using a computationally intensive Monte Carlo approach.

**Results:**

Our recovery approach use survey data from the population targeted for an estimation of tree biomass. Examples from Germany and Mexico illustrate and validate the methods. Applications with biomass estimation and robust recovered fit statistics gave reasonable estimates of model errors in tree level estimates of biomass.

**Conclusions:**

It is good practice to provide estimates of uncertainty to any model-dependent estimate of above ground biomass. When a direct approach to estimate uncertainty is impossible due to missing model statistics, the proposed robust procedure is a first step to good practice. Our recommended approach offers protection against inflated estimates of precision.

## Background

The importance of forest biomass for the global carbon cycle is widely recognized [1–4]. The imperative of maintaining global levels of forest biomass and slowing regional rates of decline [5] has fostered international cooperation, initiatives, and projects to this end [6–8].

A large number of countries have agreed to implement an accounting system for forest carbon and to report on national-level annual gains and losses [9–11].

With few exceptions, the forest carbon accounting system has a national forest inventory at its core, and a suite of models to expand and transform inventory data to forest carbon [12–14]. Carbon components not fully covered by an inventory are typically estimated from activity data (e.g. harvest, disturbance, and erosion) and models fitted to data from research studies of, for examples: litter-fall; litter-decomposition; fine-root turnover; seed production; and dead and downed-woody debris.

An estimate of the uncertainty in a carbon balance has become a routine requirement [15, 16]. When the core inventory data comes from a probability sample, the uncertainty arises from three sources: observational and measurement errors [17–19], sampling errors, and errors in model parameters [12, 20]. The live above-ground forest tree biomass (AGB) accounts for the largest contribution to the forest carbon balance [21, 22].

In situ determination of AGB is extremely costly and destructive. A model‒dependent approach with prediction of biomass from a biomass equation, with easy-to-measure explanatory variables, is the only practically feasible alternative [21, 23].

The development of a complete set of regional, species- and stratum-specific biomass models constitutes a heavy financial outlay that cannot be met in many parts of the world. As a substitute for models fitted to local data, an analyst may decide to use the most suitable off-the-shelf biomass equation [21, 23–36].

It is, of course, very difficult to ascertain whether an off-the-shelf model is suitable for a particular application or not [37]. It remains a risky proposition to use externally fitted models without any form of validation or re-calibration to local conditions [38]. An adopted model generates the desired predictions of above-ground biomass but a valid estimate of the associated covariance of model-parameters is needed to compute an estimate of the uncertainty in a prediction [12, 39, p. 73, 40]. A model-bias can only be quantified in a validation with actual observations of above-ground biomass and the predictors in a model [41, pp. 172 and 232, 42].

Although we have a plethora of equations for above-ground biomass as a function of, for example, stem diameter at a reference height of 1.3 m above ground level [21, 26, 31, 43], information regarding the covariance matrix of model parameters is often missing. Available fit statistic is generally limited to one or more of the following: standard errors of estimated parameters, the coefficient of determination, the standard deviation of lack-of-fit residuals, and sample size [44].

This study demonstrates methods for recovering a covariance matrix for model parameters in a biomass equation from fit statistics restricted to: sample size (*n*) and the coefficient of determination (R^2^) [44]. Our non-use of a possibly available estimate of the standard deviation of empirical residuals rests with its sensitivity to outliers [45], a strong dependency on the sampling design [39, p. 55], the distribution of the response and explanatory variables in the study that gave us the equation of interest. Wayson et al. [44] proposed a Monte-Carlo approach to recover missing estimates of the covariance among parameters in a biomass equation. The key idea in their approach is to generate a distribution of pseudo-data that mirrors, to the extent possible, a known or assumed distribution of explanatory variables in the sample trees behind an equation. The tenet behind our approach is different. It is rooted in survey sampling [39]. Hence, the recovered estimates of uncertainty are assumed compatible with estimates that could have been obtained from a sample taken from the population, for which we desire estimates of biomass. It is fully recognized that our recovery is neither perfect nor unbiased. However, supported by our results, we argue that our approach is consistent with the main objective of any recovery procedure: to estimate model errors in population estimates of biomass as opposed to a rediscovery of ‘lost’ estimates of model errors.

Our demonstrations include examples with equations and data from the first German national forest inventory in 1987 (BWI-1) [40, 46] and the 2004–2009 Mexican National Forest Inventory [47–49]. We discuss limitations to our approach, and recommend a robust recovery method. We also emphasize the need to develop new and fully documented biomass equations for important species in regions where they are currently lacking.

## Results

### Examples from Germany

Substitutes for missing covariance matrices for the biomass models in Table 1 are listed in Table 2. For refitted matrices, there were three rejections of the null hypothesis of equality (actual = refitted) at the 5 % level of significance. For the recovered matrices there were one rejection, and for the robust recovery there were zero rejections. A distinct pattern emerged when comparing refitted, recovered, and robust variances. Refitting appears to overestimate the variance in a regression parameter; by approximately 70 % for the first parameter and approximately 35 % for the second parameter. In contrast, the recovered variances were, on average, smaller than the actual variances (5 and 16 %, respectively). Robust estimates of variance were closer to actual estimates of variance than refitted and recovered estimates. Substitute covariance matrices for the nonlinear models were, in general, closer to the missing (actual) covariance matrix than a substitute covariance matrix for a linear model.

**Table 1:** Species group above-ground forest tree biomass (AGB kg tree^−1^) equations

Species	#	Equation $\widehat{{\text{AGB}}} =$	$\hat{R}^{2}$	$\hat{\sigma }_{e}^{2}$ 10^3^ kg	Wts
BEECH^a^	1	$0.901{\text{DBH}}^{2} - 6.382\sqrt {\text{DBH} \times {\text{HT}}}$	0.96	75.3	n.a.
	2	$\left( {9.645{\text{DBH}} - 0.648\sqrt {\text{HT}} } \right)^{2}$	0.95	79.2	n.a.
SPRUCE^a^	5	$0.447{\text{DBH}}^{2} - 1.189\sqrt {\text{DBH} \times {\text{HT}}}$	0.98	18.7	n.a.
	6	$\left( {0.634{\text{DBH}} - 0.426\sqrt {\text{HT}} } \right)^{2}$	0.98	18.2	n.a.
PINE^a^	9	$0.450{\text{DBH}}^{2} - 0.014\sqrt {\text{DBH} \times {\text{HT}}}$	0.98	14.5	n.a.
	10	$0.658{\text{DBH}}^{2} - 0.124\sqrt {\text{DBH} \times {\text{HT}}}$	0.98	14.5	n.a.
BEECH	3	$0.887{\text{DBH}}^{2} - 6.487\sqrt {\text{DBH} \times {\text{HT}}}$	0.96	74.4	DBH^−2^
	4	$\left( {10.127{\text{DBH}} - 1.167\sqrt {\text{HT}} } \right)^{2}$	0.95	75.3	DBH^−2^
SPRUCE	7	$0.492{\text{DBH}}^{2} - 1.409\sqrt {\text{DBH} \times {\text{HT}}}$	0.97	19.0	DBH^−2^
	8	$\left( {0.689{\text{DBH}} - 0.064\sqrt {\text{HT}} } \right)^{2}$	0.97	18.7	DBH^−2^
PINE	11	$0.479{\text{DBH}}^{2} - 1.927\sqrt {\text{DBH} \times {\text{HT}}}$	0.97	14.7	DBH^−2^
	12	$0.690{\text{DBH}}^{2} - 0.205\sqrt {\text{DBH} \times {\text{HT}}}$	0.97	14.5	DBH^−2^
BEECH^b^	13	$Exp\left[ {0.006 + 10.933\,\frac{\text{DBH}}{\text {DBH} + 21.216}} \right]$	0.99^c^	n.a.	n.a.
SPRUCE^b^	14	$Exp\left[ { - 1.694 + 10.825\frac{\text{DBH}}{\text{DBH} + 11.816}} \right]$	0.99^c^	n.a.	n.a.
PINE^b^	15	$Exp\left[ { - 2.688 + 10.745\,\frac{\text{DBH}}{\text{DBH} + 8.062}} \right]$	0.99^c^	n.a.	n.a.

Sample size is 50 trees per species group.

^a^BWI-1987 Predictions of AGB times a uniformly distributed random error [0.9,1.1] fitted to DBH (mm) and HT (dm).

^b^Generalized AGB equations from Table 8 (Temperate zone) in Muukkonen P and Heiskanen J [76]. DBH is in centimeters (cm).

^c^Amount of variation in predicted AGB values captured by the generalized equation.

Data were selected from 335 plots from the 1987 (West) German national forest inventory (BWI-1987). Plots were dominated by one of the three species groups. Selected trees have a DBH ≥ 7 cm, and were selected with a probability proportional to their basal area at breast height (basal area factor of 4), [77, ch. 8].

**Table 2:** Actual, refitted, and recovered covariance matrices of non-weighted regression coefficients in equations

Eq #	Actual	Refitted	Recovered	Recovered (robust)	*P*_*1*_ _×*100*_	*P*_*2*×*100*_	*P*_*3*_ _×*100*_
1	$$\left( {\begin{array}{*{20}c} {2.62 \times 10^{ - 3} } & { - 0.132} \\ { - 0.132} & {8.67} \\ \end{array} } \right)$$	$$\left( {\begin{array}{*{20}c} {3.30 \times 10^{ - 3} } & { - 0.179} \\ { - 0.179} & {11.4} \\ \end{array} } \right)$$	$$\left( {\begin{array}{*{20}c} {2.75 \times 10^{ - 3} } & { - 0.154} \\ { - 0.154} & {10.2} \\ \end{array} } \right)$$	$$\left( {\begin{array}{*{20}c} {3.01 \times 10^{ - 3} } & { - 0.167} \\ { - 0.167} & {10.9} \\ \end{array} } \right)$$	0.5	64	68
2	$$\left( {\begin{array}{*{20}c} {0.267} & { - 0.746} \\ { - 0.746} & {2.25} \\ \end{array} } \right)$$	$$\left( {\begin{array}{*{20}c} {0.316} & { - 0.871} \\ { - 0.871} & {2.53} \\ \end{array} } \right)$$	$$\left( {\begin{array}{*{20}c} {0.240} & { - 0.677} \\ { - 0.677} & {2.33} \\ \end{array} } \right)$$	$$\left( {\begin{array}{*{20}c} {0.264} & { - 0.740} \\ { - 0.740} & {2.19} \\ \end{array} } \right)$$	99	95	98
5	$$\left( {\begin{array}{*{20}c} {1.14 \times 10^{ - 3} } & { - 0.605 \times 10^{ - 1} } \\ { - 0.605 \times 10^{ - 1} } & {3.56} \\ \end{array} } \right)$$	$$\left( {\begin{array}{*{20}c} {1.39 \times 10^{ - 3} } & { - 0.598 \times 10^{ - 1} } \\ { - 0.598 \times 10^{ - 1} } & {3.00} \\ \end{array} } \right)$$	$$\left( {\begin{array}{*{20}c} {1.02 \times 10^{ - 3} } & { - 0.436 \times 10^{ - 1} } \\ { - 0.436 \times 10^{ - 1} } & {2.21} \\ \end{array} } \right)$$	$$\left( {\begin{array}{*{20}c} {1.08 \times 10^{ - 3} } & {0.465 \times 10^{ - 1} } \\ {0.465 \times 10^{ - 1} } & {2.35} \\ \end{array} } \right)$$	4	6	7
6	$$\left( {\begin{array}{*{20}c} {1.47 \times 10^{ - 1} } & { - 0.383} \\ { - 0.383} & {1.04} \\ \end{array} } \right)$$	$$\left( {\begin{array}{*{20}c} {2.00 \times 10^{ - 1} } & { - 0.469} \\ { - 0.469} & {1.15} \\ \end{array} } \right)$$	$$\left( {\begin{array}{*{20}c} {1.38 \times 10^{ - 1} } & { - 0.319} \\ { - 0.319} & {0.785} \\ \end{array} } \right)$$	$$\left( {\begin{array}{*{20}c} {1.46 \times 10^{ - 1} } & { - 0.338} \\ { - 0.338} & {0.831} \\ \end{array} } \right)$$	5	36	36
9	$$\left( {\begin{array}{*{20}c} {5.53 \times 10^{ - 4} } & { - 0.322 \times 10^{ - 1} } \\ { - 0.322 \times 10^{ - 1} } & {2.19} \\ \end{array} } \right)$$	$$\left( {\begin{array}{*{20}c} {1.33 \times 10^{ - 3} } & { - 0.636 \times 10^{ - 1} } \\ { - 0.636 \times 10^{ - 1} } & {3.44} \\ \end{array} } \right)$$	$$\left( {\begin{array}{*{20}c} {5.61 \times 10^{ - 4} } & { - 0.273 \times 10^{ - 1} } \\ { - 0.273 \times 10^{ - 1} } & {1.50} \\ \end{array} } \right)$$	$$\left( {\begin{array}{*{20}c} {6.05 \times 10^{ - 4} } & { - 0.293 \times 10^{ - 1} } \\ { - 0.293 \times 10^{ - 1} } & {1.61} \\ \end{array} } \right)$$	4	3	5
10	$$\left( {\begin{array}{*{20}c} {7.84 \times 10^{ - 2} } & { - 0.227} \\ { - 0.227} & {0.697} \\ \end{array} } \right)$$	$$\left( {\begin{array}{*{20}c} {1.27 \times 10^{ - 1} } & { - 0.499} \\ { - 0.499} & {0.86} \\ \end{array} } \right)$$	$$\left( {\begin{array}{*{20}c} {8.20 \times 10^{ - 2} } & { - 0.210} \\ { - 0.210} & {0.561} \\ \end{array} } \right)$$	$$\left( {\begin{array}{*{20}c} {8.82 \times 10^{ - 2} } & { - 0.225} \\ { - 0.225} & {0.599} \\ \end{array} } \right)$$	12	15	19

Actual covariance matrices are based on a sample size of 50. *P*_i_ (*i* = 1, 2, 3) is the probability under *H*_0_ of: (1) actual = refitted; (2) actual = recovered; (3) actual = robust. See Table 1 for reference to equation numbering.

Taking into consideration that the relative error in the regression coefficient to diameter DBH or DBH^2^ is six to twelve times smaller than the relative error in the regression coefficient to $\sqrt {\text{DBH} \times {\text{HT}}} \;{\text{or}}\;\sqrt {\text{HT}}$, a bias in the former is much more serious than in the latter. For un-weighted linear and nonlinear equations, the robust procedure appears as the most attractive. As well, the strong impact of errors in the first regression coefficient on a tree-level estimate of AGB amplifies concerns surrounding the overestimation of model-errors encountered with the refitting procedure.

For the weighted least squares equations, the estimates of the substitute covariance matrix are in Table 3. Generally the results were worse entailing larger differences between estimated substitutes and actual covariance matrices. The refitted matrices were worst with five out of six significant departures from the actual matrices, and in terms of seriously overestimating the variances. The best results were obtained with the recovered matrices (two rejections of the null hypothesis of no difference). Yet there is an average overestimation of the first variance by 23 % and an average underestimation of the second by 25 %. Considering the larger contribution to the model error variance from the former, the overestimation is a concern. A robustly recovered matrix was in four cases significantly different from the actual covariance matrix and overestimated variances by 72 and 24 %.

**Table 3:** Actual, refitted, and recovered covariance matrices of regression coefficients in weighted least squares equations in Table 1

Eq. #	Actual	Refitted	Recovered	Recovered (robust)	*P*_*1*_ _×*100*_	*P*_*2*_ _×*100*_	*P*_*3*_ _×*100*_
3	$$\left( {\begin{array}{*{20}c} {2.62 \times 10^{ - 3} } & { - 0.132} \\ { - 0.132} & {8.67} \\ \end{array} } \right)$$	$$\left( {\begin{array}{*{20}c} {2.15 \times 10^{ - 3} } & { - 0.249} \\ { - 0.249} & {4.11} \\ \end{array} } \right)$$	$$\left( {\begin{array}{*{20}c} {2.66 \times 10^{ - 3} } & { - 0.0995} \\ { - 0.0995} & {5.00} \\ \end{array} } \right)$$	$$\left( {\begin{array}{*{20}c} {3.54 \times 10^{ - 3} } & { - 0.175} \\ { - 0.175} & {10.4} \\ \end{array} } \right)$$	0.1	0.2	0.0
4	$$\left( {\begin{array}{*{20}c} {0.268} & { - 0.747} \\ { - 0.747} & {2.25} \\ \end{array} } \right)$$	$$\left( {\begin{array}{*{20}c} {0.203} & { - 1.60} \\ { - 1.60} & {1.26} \\ \end{array} } \right)$$	$$\left( {\begin{array}{*{20}c} {0.300} & { - 0.723} \\ { - 0.723} & {1.90} \\ \end{array} } \right)$$	$$\left( {\begin{array}{*{20}c} {0.493} & { - 1.28} \\ { - 1.28} & {3.51} \\ \end{array} } \right)$$	13	8	0.0
7	$$\left( {\begin{array}{*{20}c} {0.816 \times 10^{ - 3} } & { - 0.0336} \\ { - 0.0336} & {1.66} \\ \end{array} } \right)$$	$$\left( {\begin{array}{*{20}c} {3.78 \times 10^{ - 3} } & { - 0.115} \\ { - 0.115} & {4.37} \\ \end{array} } \right)$$	$$\left( {\begin{array}{*{20}c} {1.36 \times 10^{ - 3} } & { - 0.0417} \\ { - 0.0417} & {1.61} \\ \end{array} } \right)$$	$$\left( {\begin{array}{*{20}c} {0.578 \times 10^{ - 3} } & { - 0.0251} \\ { - 0.0251} & {1.27} \\ \end{array} } \right)$$	1	0.8	29
8	$$\left( {\begin{array}{*{20}c} {0.139} & { - 0.328} \\ { - 0.328} & {0.813} \\ \end{array} } \right)$$	$$\left( {\begin{array}{*{20}c} {0.521} & { - 1.01} \\ { - 1.01} & {2.11} \\ \end{array} } \right)$$	$$\left( {\begin{array}{*{20}c} {0.192} & { - 0.381} \\ { - 0.381} & {0.82} \\ \end{array} } \right)$$	$$\left( {\begin{array}{*{20}c} {0.297} & { - 0.630} \\ { - 0.630} & {1.41} \\ \end{array} } \right)$$	0.0	7	4
11	$$\left( {\begin{array}{*{20}c} {5.53 \times 10^{ - 4} } & { - 0.0322} \\ { - 0.0322} & {2.19} \\ \end{array} } \right)$$	$$\left( {\begin{array}{*{20}c} {2.13 \times 10^{ - 3} } & { - 0.0778} \\ { - 0.0778} & {3.46} \\ \end{array} } \right)$$	$$\left( {\begin{array}{*{20}c} {5.78 \times 10^{ - 4} } & { - 0.0213} \\ { - 0.0213} & {0.959} \\ \end{array} } \right)$$	$$\left( {\begin{array}{*{20}c} {1.40 \times 10^{ - 3} } & { - 0.0549} \\ { - 0.0549} & {2.30} \\ \end{array} } \right)$$	0.0	16	0.5
12	$$\left( {\begin{array}{*{20}c} {7.84 \times 10^{ - 2} } & { - 0.227} \\ { - 0.227} & {0.698} \\ \end{array} } \right)$$	$$\left( {\begin{array}{*{20}c} {0.153} & { - 0.342} \\ { - 0.342} & {0.813} \\ \end{array} } \right)$$	$$\left( {\begin{array}{*{20}c} {8.96 \times 10^{ - 2} } & { - 0.202} \\ { - 0.202} & {0.485} \\ \end{array} } \right)$$	$$\left( {\begin{array}{*{20}c} {0.202} & { - 0.457} \\ { - 0.457} & {1.06} \\ \end{array} } \right)$$	0.0	38	4

Actual covariance matrices are based on a sample size of 50. *P*_i_ (*i* = 1, 2, 3) is the probability under *H*_0_ of: (1) actual = refitted; (2) actual = recovered; (3) actual = robust.

Recovering an estimate of the residual variance was, as expected, easier than recovering a covariance matrix. The relative error in recovered estimates of the residual standard error varied from approximately −20 to +35 %. Two of eight estimates were significantly different from the actual values (*F*-ratio test, *P* = 0.02), for the remaining six, the level of significance was 0.10 or greater.

Attempts at a recovery of the covariance matrices for the generalized above-ground biomass Eqs. 13–15 in Table 1 [26] failed, regardless of method. With the recovery methods, the estimated standard deviations of the three regression parameters were 2–8 times greater than those listed in Table 3 of Muukkonen [26]. Had we used the tabled values of the root mean squared error in lieu of the recovered substitute, the estimated errors would have been approximately 30–70 times too small. The failure is easy to explain: the fit-statistics of the generalized model apply to the set of models that are generalized. Footnotes to Table 3 in Muukkonen [26] carefully explain the constrained interpretation of the table entries. Due to the poor accuracy of the recovered generalized covariance matrices they were not used to gauge the error-propagation to estimates of tree-level AGB.

All recovery procedures are fraught with numerical problems due to co-linearity among regression coefficients (correlations coefficients varied between −0.87 and −0.97), and large differences in accuracy of parameter estimates. For example, the matrix condition number varied between 14.1 and 14.9, and determinants were less than 10^−5^ suggesting a serious potential of amplified estimation errors when inverting a covariance matrix [50, 51]. Challenges of this nature will also be encountered in applications of the proposed procedures.

A summary of the effect of replacing a missing (actual) covariance matrix with a substitute approximation on the model-error in an estimate of the mean per tree AGB (kg) is provided in Table 4. With the un-weighted linear models the relative model error in the average tree-level estimate of AGB is 7–12 % (column ACT in Table 4). Model errors in estimates based on a nonlinear un-weighted equation were approximately 2–3 % points lower.

**Table 4:** Relative model errors (%) in estimates of the mean per tree above-ground tree biomass with actual (ACT), refitted (REFIT), recovered (RECOV), and robustly recovered (RREC) covariance matrices for the parameters in the biomass equations in Table 1

Species	Model	Weights?	ACT	REFIT	RECOV	RREC
BEECH	LIN	No	11.5	14.0	12.3	12.8
	NLIN	No	8.7	9.1	8.0	8.4
	LIN	Yes	4.7	8.8	7.8	11.6
	NLIN	Yes	4.4	6.6	6.9	9.4
SPRUCE	LIN	No	9.8	8.4	7.2	7.4
	NLIN	No	7.0	6.6	5.4	5.6
	LIN	Yes	6.2	10.0	5.4	7.4
	NLIN	Yes	5.6	8.5	4.8	6.6
PINE	LIN	No	6.9	5.9	4.9	5.1
	NLIN	No	5.3	4.7	3.8	4.0
	LIN	Yes	4.8	22.3	3.5	4.3
	NLIN	Yes	4.2	19.0	3.0	3.8

Weighted regressions were uniformly superior with the lowest relative errors. Results with the substitute covariance matrices followed—by and large—these trends with estimates within one to 6 % points from results with the actual estimate of covariance. In the case of weighted regressions: two poor results with the refitting procedure with PINE data, and two for the robust recovery with BEECH data, stands out as examples of inflated estimates of model-error. The remaining estimates of error appear reasonable; yet do not indicate that one recovery procedure is substantially and consistently better than the presented alternatives.

### Examples from Mexico

For *Guazuma ulmifolia* and *Ochroma pyramidale* the substitute estimates of the parameter error variances were, not statistically significant from the actual estimates of error (Table 5). This is spite of overestimating, by a factor of approximately two, the variances in the regression parameters for *G*. *ulmifolia*. The relative small sample sizes of 18 and 16 trees limit our power to declare practically important differences significant. In case of *Inga vera* and *Trichospernum mexicanum* the substitute variances were two to four times larger than the published estimates. Each recovery procedure led to inflated estimates of variance. The basic recovery method holds a slight edge over the other two. We did not attempt a weighting scheme in the recovery procedure as the log transformation of AGB and DBH in most cases remove variance heteroscedasticity in the original scale of the residuals. Power functions as used for *Quercus* spp. are extremely sensitive to the weighting schemes used in the German examples. Besides, the original biomass equations were not obtained by weighted least squares [52] so we did not employ a weighted recovery scheme.

**Table 5:** Actual, refitted, and recovered variances of regression coefficients in Eqs. 1–7 in Table 8

Eq #	Actual	Refitted	Recovered	Recovered (robust)	*P*_*1*_ _×*100*_	*P*_*2*_ _×*100*_	*P*_*3*_ _×*100*_
1	(0.04, 0.01)	(0.08, 0.01)	(0.07, 0.01)	(0.10, 0.02)	56	74	49
2	(0.55, 0.42, 0.020)	(2.64, 1.39, 0.044)	(2.36, 1.25, 0.039)	(2.88, 1.52, 0.048)	0.2	0.1	0.1
3	(0.27, 0.048)	(0.29, 0.041)	(0.26, 0.039)	(0.35, 0.053)	99	98	96
4	(0.084, 0.017)	(0.33, 0.049)	(0.29, 0.043)	(0.37, 0.06)	2	5	1
5	n.a.	(0.32, 7.1)10^−3^	(0.20, 9.4)10^−3^	(0.23, 10.6)10^−3^	n.a.	n.a.	n.a.
6	n.a.	(0.61, 4.7)10^−1^	(0.42, 32.2)10^−2^	(0.12, 9.0)10^−1^	n.a.	n.a.	n.a.
7	n.a.	(0.98, 16.8)10^−3^	(0.29, 19.4)10^−3^	(0.74, 29.4)10^−3^	n.a.	n.a.	n.a.

Actual covariance matrices are based on sample sizes listed in Table 8. *P*_i_ (*i* = 1, 2, 3) is the probability under *H*_0_ of: (1) actual = refitted; (2) actual = recovered; (3) actual = robust.

Substitute estimates of the residual standard error were considerably and statistically significantly smaller (30–240 %) than the published values. These results paired with the inflation of the variance of regression coefficients suggest a much smaller variation of the explanatory variables in the samples from the national inventory than in the sample used for fitting. A uniform distribution of the explanatory variables in the model fitting sample [53] could explain our results.

Tabled estimates of the residual standard errors for the three *Quercus* spp. Were three to four times smaller than recovered estimates. We noted that even a small reduction of 1–2 % in the published value of $\hat{R}^{2}$ would bring the two sets of estimates within approximately 20 % of each other. Power functions are notorious in this regard.

When the uncertainty in biomass equation parameters was propagated to tree-level estimate of AGB, we obtained the average relative per tree model-errors in Table 6. Overall, the relative model errors in the average per tree AGB in *G*. *ulmifolia* appears too low, despite an apparent overestimation of the errors in the model parameters. Refitting of a missing covariance matrix via the parametric bootstrapping generated unrealistic large estimates of relative errors in *Quercus laeta* and *Quercus* spp. Numerical instability of the covariance matrix, small sample sizes, and random multiplicative residuals with a large variance is a recipe for poor results. As expected, the robust recovery produces the largest estimates of relative errors.

**Table 6:** Estimates of mean AGB kg tree^−1^ and relative errors (%) in estimates in mean AGB for seven Mexican species

Species	$\hat{\text{AGB}}$	REFIT	RECOV	RREC
*Guazuma ulmifolia*	54	5.0	4.7	5.4
*Inga vera*	198	8.6	8.2	9.0
*Ochroma pyramidale*	50	11.8	11.2	12.8
*Trichospernum mexicanum*	51	13.9	13.1	15.1
*Quercus castenea*	75	23.2	28.6	33.9
*Quercus latea*	129	101.7	23.9	30.7
*Quercus* spp.	135	53.0	13.1	13.8

Estimates are based on tree data provided by the Mexican NFI (see Table 9). The errors are derived with refitted (REFIT), recovered (RECOV), and robustly recovered (RREC) covariance matrices for the parameters in the biomass equations in Table 8.

## Discussion

The need for forest biomass equations has increased sharply over the past decades in response to efforts directed at quantifying stock and stock-changes in forest carbon and the potential for bioenergy extraction [21, 42, 54]. Ideally there would be an equation for each tree species and region with distinct growth forms and management regimes [55, 56]. We are still far from this ideal. Even the equations we have are generally based on very limited sampling within a relatively small area and range of tree sizes [21]. This is understandable in light of the high costs of producing a biomass equation [21, 26, 57]. Biomass estimates for large trees are therefore fraught with problems of applicability of available biomass equations.

In the computation of forest biomass in a large region, country, or even a continent, it is common practice to use a suitable biomass equation for a particular species and growth region [58–60]. In most cases, there is no separate calibration of chosen biomass equations.

On this background, national and regional estimates of above-ground biomass should be regarded as no more than first-order approximations [16]. The requirement [11] to quantify or at least assess uncertainty in a national or regional estimate of forest biomass has precipitated a need for estimates of errors in the parameters of employed biomass equations. For a large number of equations, this information is partially or entirely missing [31, 44].

In a context of model-dependent estimation of forest tree biomass and model-errors in these estimates, a covariance matrix of the model parameters is needed [12, 40]. When this statistic is missing a substitute is needed. Wayson et al. [44] proposed a computationally intensive method for generating a large number of pseudo data of the dependent and independent variables in a biomass equation. Samples are then drawn repeatedly and the model is refitted each time. The sampling aims at mimicking the actual sampling process (if known) of the original data behind a biomass equation.

Our proposed procedures for computing a substitute for a missing covariance matrix are computationally faster and make direct use of data of the explanatory variables sampled from the population targeted for an estimation of biomass. The distribution of the explanatory variables used to compute (recover) a covariance matrix plays a pivotal role in both approaches. If the actual distribution behind an equation differs from the distribution in the recovery process, a covariance matrix different from the actual (but unknown) will emerge from a recovery procedure. We saw several examples of this in our examples, but an equal number of examples where a substitute matrix was not statistically different from the target matrix. Wayson et al. [44] do not report at this level of details, but we surmise that they encountered similar issues. It is now a question of whether these differences are relevant or not. We argue, that sampling the explanatory variables from the target population vouch for estimates adapted to the application domain rather than to a small sample of trees with unknown representation in the target population.

The most intuitive approach to recover a missing covariance matrix is a variant of the parametric bootstrap [61]. In the textbook version of a parametric bootstrap, *n* pseudo observations of *Y* are generated a large number of times (say *B*) by adding a random draw from the observed empirical regression residuals to the *n* model predictions obtained from the original regression model and the observed explanatory variables. The regression model is then refitted *B* times to the pseudo observations of *Y*. At the end, the analyst has *B* replications of the covariance matrix of the model parameters. Without observed residuals, this approach is not feasible. Instead, our recovery by refitting resorted to random sampling of the explanatory variables from the target population for biomass estimation, and residuals from a distribution deemed realistic to the case at hand (e.g. a gamma distribution for multiplicative residuals). Although this method in many cases was as good as with alternative approaches, it was equally clear that it entails a considerable risk of poor results. A risk traced to random interactions between residuals and the explanatory variables. For that reason we do not recommend a recovery by model refitting with simulated residuals.

The examples from Germany confirmed that in presence of heteroscedasticity in the model residuals, a weighting with the inverse to the presumed residual variance can be effective [62, ch. 2.11, 63, ch. 2.1]. To carry this efficiency through to a recovered covariance matrix, a weighting scheme applied to the original biomass equation should be replicated in a recovery procedure.

A matrix recovery based on the average (vector) gradient of the model parameters with respect to the explanatory variables was, in the balance, better suited for the purpose of estimation of model errors in tree-level biomass estimates. A robust variant of the recovery is easy to compute and—despite expected and observed larger estimates of model-errors—we recommend this procedure as a prudent choice. For the purpose of reasonable estimates model-errors in tree-level estimates of biomass, it is not a strict requirement that a recovered covariance matrix is close to the actual but missing matrix. Most of our estimates, but especially those obtained with the robust recovery procedure, seem reasonable [13, 16, 20, 57, 64]. Our resampling of explanatory variables from inventory data representing the population targeted for an estimation of biomass, ensures that the mean of the explanatory variables will be close to the mean in the target population. *Ceteris paribus*, this will counter the aforementioned inflation of model-parameter variances [39, ch. 5.4].

An attempt to recover a covariance matrix can end in failure. A failure was demonstrated with the generalized biomass equations for beech, pine, and spruce in the temperate zone [26, Table 1]. A failure is pre-ordained when estimates of R^2^ and a root mean squared errors are incompatible with the biomass equation applied to actual data. Our experience should raise awareness of potential pitfalls in published fit-statistics for a generalized equation, unless they reflect a proper meta-analysis [65].

Throughout we have treated published fit statistics as known entities. It would have been preferable to consider an empirical Bayesian recovery procedure [66]. The coefficient of determination is pivotal in our proposed procedures. Its sampling variance can only be estimated from the data supporting a biomass equation [67]. To recognize sampling variance in *R* ^2^, a recovery is repeated a large number of times, each with a random draw from an anticipated distribution of *R* ^2^, to create an empirical Bayes posterior distribution of the recovered statistic. The recovery procedure by Wayson et al. [44] contains elements of a Bayesian approach.

Although a recovered covariance matrix affords an estimate of the model error in a tree level biomass estimate, the model-error is conditional on a correctly specified model. If a published biomass equation is the result of an intensive model and variable screening process, we must expect optimism in published statistics and model-bias [68].

We have demonstrated the recovery of a missing covariance matrix without too much concern about sample size. Clearly, a biomass equation derived from a small sample size has a relatively high risk of model bias due to a high influence of individual observations [62, p. 170]. It is not possible to give a definite recommendation about the minimum sample size for our robust recovery procedure. However, a first approximation can be gained from the following example: If we have fitted a linear regression model with three parameters, and we wish to declare a standardized regression residual of 3 as significant at the 5 % level (an indication that the model is unduly influenced by residuals of this magnitude), we need a sample size of approximately 55 [69]. Thus an application of our recovery procedure for regression models supported by less than 55 trees should proceed with caution and attention to robustness.

In large sample inventories the model errors in point estimates of biomass will often dominate sampling errors [12, 40]. Fortunately, when estimating a temporal change in biomass and carbon stock between two inventories, model errors in a difference all but cancel [Ibid]. Thus applying recovered conservative (robust) estimates of a missing covariance matrix will have little impact on the estimate of model errors in a difference.

We have demonstrated that reasonable (robust) estimates of model-errors in estimates of tree-level biomass can be derived from a minimum of two available fit statistics for a biomass equation: the coefficient of determination, and sample size. To complete an estimation of model-errors an analyst need access to forest inventory sample data of the explanatory variables from the population targeted for biomass estimation.

## Conclusions

It is good practice to provide estimates of uncertainty to any model-dependent estimate of above ground biomass. When a direct approach to estimate uncertainty is impossible due to missing model statistics, the proposed robust procedure is a first step to good practice. Our recommended approach offers protection against inflated estimates of precision.

## Methods

### The biomass model

The model we consider for above-ground live tree biomass is parametric and can be expressed as

1 $$y_{i} = f\left( {{\mathbf{x}}_{i} ; {\mathbf{b}}} \right) + e_{i}$$

where *y*_*i*_ is the above-ground forest tree biomass (AGB in kg) of the *i*th tree, *f* is a known function (linear or nonlinear), **x**_*i*_ is a *p* × 1 row vector of regressor variables including an intercept (if any), **b** is a *q* × *1* vector of model parameters, and *e*_*i*_ is a residual error. For a linear model *p* = *q*.

A model *f* fitted to *n* observations of **x**_*i*_ and *y*_*i*_(*i* = 1,…, *n*) allows a prediction of the expected biomass in the, say, *j*th tree $\left( {\hat{y}_{j} } \right)$ from knowledge of **x**_*j*_ and the estimated parameters ${\hat{\mathbf{b}}}$. In the application context of a forest inventory (survey) the model in (1) is used to predict AGB for out-of-sample trees. An estimator of the approximate out-of-sample model error variance in an estimate of AGB for a tree *j* with a known (measured) vector **x**_*j*_ of explanatory variables is [70, ch. 6.3]

2 $$\hat{V}\left( {\hat{{\text{AGB}_{j} }}} \right) = \hat{\sigma }_{e}^{2} + \frac{{\partial f\left( {{\mathbf{x}}_{j} {\mathbf{|b}}} \right)}}{{\partial {\mathbf{b}}}}^{t} \hat{\text{cov}}\left( {{\hat{\mathbf{b}}}} \right)\frac{{\partial f\left( {{\mathbf{x}}_{j} {\mathbf{|b}}} \right)}}{{\partial {\mathbf{b}}}}$$

where $\hat{\sigma }_{e}^{2}$ is an estimate of the variance of lack-of-fit residuals (*e*_*i*_) of the trees used to fit the model in (1), and ∂*f* (**x**_*j*_|**b**)∂^−1^ **b** is the vector of derivatives (gradients) with respect to the model parameters, and $\hat{\text{cov}}\left( {{\hat{\mathbf{b}}}} \right)$ is an estimate of the covariance among model parameters. All gradients are evaluated at the least squares estimate of **b**. A superscript ‘*t*’ denotes the transpose of a vector or a matrix. When the model is linear in **b** the derivatives in 2 reduces to the vector **x**.

The *q* × *q* covariance matrix for ${\hat{\mathbf{b}}}$ is [63, p. 17]

3 $${\text{c}}\hat{\text{o}}{\text{v}}\left( {\mathbf{b}} \right) = \hat{\sigma }_{e}^{2} \left( {{\hat{\mathbf{F}}}^{t} {\hat{\mathbf{F}}}} \right)^{ - 1} \quad {\text{with}}\quad {\hat{\mathbf{F}}} = \left\{ {\frac{{\partial f\left( {{\mathbf{x}}_{1} |{\mathbf{b}}} \right)}}{{\partial {\mathbf{b}}}}, \ldots ,\frac{{\partial f\left( {{\mathbf{x}}_{i} |{\mathbf{b}}} \right)}}{{\partial {\mathbf{b}}}}, \ldots ,\frac{{\partial f\left( {{\mathbf{x}}_{n} |{\mathbf{b}}} \right)}}{{\partial {\mathbf{b}}}}} \right\}^{t} \,$$

### The estimation problem

It is clear from (2) that we cannot estimate the error in an out-of-sample estimate of the AGB in a single tree unless we have reasonable estimates of $\hat{\sigma }_{e}^{2}$ and $\hat{\text{cov}}\left( {{\hat{\mathbf{b}}}} \right)$. Note, when we wish to estimate the error in an average of AGB in a large number (*m*) of trees, the contribution to the error from the residual variance can be ignored as it declines at a rate of *m* ^−1^, the second term, however, is only averaged over *m* [62, pp. 28–30].

When we are tasked with estimating the error variance in (2) but do not have estimates of $\hat{\sigma }_{e}^{2}$ or $\hat{\text{cov}}\left( {{\hat{\mathbf{b}}}} \right)$ we have to recover reasonable substitutes. Equations (2) and (3) implicitly suggest how to obtain substitutes $\tilde{\sigma }_{e}^{2}$ for $\hat{\sigma }_{e}^{2}$ and $\text{c} \widetilde{\text{o}}v\left( {{\hat{\mathbf{b}}}} \right)$ for ${\text{c}}\widetilde{\text{o}}{\text{v}}\left( {{\hat{\mathbf{b}}}} \right)$ when we at least know the sample size *n* used to estimate the parameters in the biomass model in (1), and the coefficient of determination $\hat{R}^{2}$ or, preferably, the adjusted coefficient of determination [62, p. 91].

### Recovery of missing fit statistics

A basic recovery of a substitute for $\text{c} \widetilde{\text{o}}v\left( {{\hat{\mathbf{b}}}} \right)$ begins with *B* random samples (without replacement) of size *n* of **x** taken from an inventory sample from the population for which tree-level predictions of AGB via (1) are desired. For each of the *B* samples, one first computes

4 $$\begin{aligned} \tilde{\sigma }_{e,b}^{2} &= V\left( {f\left( {{\mathbf{x}}_{b,j} |{\hat{\mathbf{b}}}} \right)} \right) \times \left( {\hat{R}^{ - 2} - 1} \right),\\ & \quad j = 1, \ldots ,n,\quad \,b = 1 \ldots ,B \end{aligned}$$

where *V*(*z*_*j*_) denotes the variance of $z_{j} = f\left( {{\mathbf{x}}_{b,j} |{\hat{\mathbf{b}}}} \right)$, and $\hat{R}^{2}$ is a known estimate of the coefficient of determination. Then ${\text{c}}\widetilde{\text{o}}{\text{v}}_{b} \left( {{\hat{\mathbf{b}}}} \right),b = 1, \ldots ,B$ is estimated as in (3). The average over the *B* replications of $\tilde{\sigma }_{e}^{2}$ and ${\text{c}}\widetilde{\text{o}}{\text{v}}\left( {{\hat{\mathbf{b}}}} \right)$ now serves to approximate the error variance in AGB of a single tree (see (2)). Implicit in this estimator of residual variance is the assumption of a homogenous error-structure.

It is clear from (4) that the estimate $\tilde{\sigma }_{e}^{2}$ depends on the sampling distribution of $f\left( {{\mathbf{x}}_{b,j} |{\hat{\mathbf{b}}}} \right)$ which may be quite different from the distribution in the original sample used in model fitting. Most biomass functions are fitted to an approximate uniform distribution of the explanatory variables, as it achieves large-sample optimality for model fitting [39, ch. 7.5]. However, for typically small sample sizes in biomass studies, this no longer holds. Our repeated sampling from the target population assuage more robust and realistic estimates of the desired covariance matrix. Albeit under the proviso that the reported coefficient of determination has not been maximized by a combination of model- and variable-selection procedures, and a sampling design that *C*. *paribus* favors a linear model.

### Recovery via refitting

A recovered estimate of the residual variance (see (4)) can be used in a parametric bootstrap [71] to recover a substitute for a missing covariance matrix $\text{c} \widetilde{\text{o}}v\left( {{\hat{\mathbf{b}}}} \right).$ The refitting begins with *n* random draws of residuals (*e*_j_^*^, *j* = 1, …, *n*) from a *t*-distribution with *n* − *q* degrees of freedom. Pseudo data $y_{j}^{*} = f\left( {{\mathbf{x}}_{j} |{\hat{\mathbf{b}}}} \right) + e_{j}^{*}$ is then used to re-estimate the parameters ${\hat{\mathbf{b}}}_{{}}^{*}$ and the associated covariance matrix $\text{c} \widetilde{\text{o}}v\left( {{\mathbf{b}}^{*} } \right)$. This process is repeated *B* times; the mean of the covariance matrices is now the substitute to use in computing an error of AGB via (2).

Adding a random residual to a biomass prediction $\hat{y}_{j}$ can make *y*_j_^*^ negative in violation of AGB ≥ 0. Should that occur we recommend computing *y*_j_^*^ from $\hat{y}_{j} \times e_{j}^{*}$ where *e*_j_^*^ is a random draw from a gamma distribution with parameters *ν* and *ν* ^−1^ (i.e. with mean 1.0 and variance *ν* ^−1^). The parameter *ν* can be found by using Goodman’s formula for the exact variance of $V\left( {\hat{y}_{i} {\kern 1pt} e_{i}^{*} } \right)$ [72]. However, in our examples this formula did not give us real-valued solutions of *ν*. By solving the equation in [5] for *ν* we obtained a good first-order approximation.

5 $$V\left( {\hat{y}_{i} {\kern 1pt} e_{i}^{*} } \right) = V\left( {\hat{y}_{i} {\kern 1pt} } \right) + \nu^{ - 1} \left( {\bar{\hat{y}}^{2} + V\left( {\hat{y}_{i} } \right)} \right)$$

### Recovery of off-diagonal elements in $\text{c} \widetilde{\text{o}}v\left( {{\hat{\mathbf{b}}}} \right)$

In some cases estimates of errors in ${\hat{\mathbf{b}}}$ are available, but without estimates of covariance. In this scenario a substitute covariance matrix can be recovered from

6 $$\begin{aligned} \text{c} \widetilde{\text{o}}v\left( {\hat{b}_{k} ,\hat{b}_{l \ne k} } \right) &= \text{c} \widetilde{\text{o}}rr\left( {\frac{{\partial f\left( {{\mathbf{x}}_{j} |{\mathbf{b}}} \right)}}{{\partial b_{k} }},\frac{{\partial f\left( {{\mathbf{x}}_{j} |{\mathbf{b}}} \right)}}{{\partial b_{l} }}} \right)_{{{\mathbf{b}} \equiv {\hat{\mathbf{b}}}}}\\ & \quad \times \sqrt {{\text{v}}\widetilde{\text{a}}{\text{r}}\left( {b_{k} } \right){\text{v}}\widetilde{\text{a}}{\text{r}}\left( {b_{l} } \right)} ,\quad k,l = 1, \ldots ,q \end{aligned}$$

### Robust recovery

A recovered substitute ${\text{c}}\widetilde{\text{o}}{\text{v}}\left( {{\hat{\mathbf{b}}}} \right)$ may differ substantially from the target covariance matrix ${\text{c}}\widetilde{\text{o}}{\text{v}}\left( {{\hat{\mathbf{b}}}} \right)$ when the distribution of **x**_*j*_ in the samples taken from the population targeted for a prediction of AGB differs from the distribution of **x**_*i*_ in the original—but unknown—sample used for model-fitting [39, ch. 5.4]. To mitigate this prospect, we propose a robust recovery of ${\text{c}}\widetilde{\text{o}}{\text{v}}\left( {{\hat{\mathbf{b}}}} \right)$. It is borrowed from Gallant AR [63] and given in (7)

7 $${\text{c}}\widetilde{\text{o}}{\text{v}}_{robust} \left( {{\hat{\mathbf{b}}}} \right) = \left( {{\hat{\mathbf{F}}}^{t} {\hat{\mathbf{F}}}} \right)^{ - 1} \left( {\sum\limits_{i = 1}^{n} {\tilde{e}_{i}^{2} \left( {\frac{{\partial f\left( {{\mathbf{x}}_{i} |{\mathbf{b}}} \right)}}{{\partial {\mathbf{b}}}}} \right)\left( {\frac{{\partial f\left( {{\mathbf{x}}_{i} |{\mathbf{b}}} \right)}}{{\partial {\mathbf{b}}}}} \right)^{t} } } \right)_{{{\mathbf{b}} \equiv {\hat{\mathbf{b}}}}} \left( {{\hat{\mathbf{F}}}^{t} {\hat{\mathbf{F}}}} \right)^{ - 1}$$

where $\tilde{e}_{i}$ is a random draw from a *t*-distribution with $\left\lfloor {0.5\,n} \right\rfloor$ degrees of freedom and variance $\tilde{\sigma }_{e}^{2}$. The choice of degrees of freedom for the *t*-distribution is arbitrary; it reflects the fact that most sample sizes supporting a tree biomass model are in the range of 6–30 [21]. A halving of these sample sizes results in increases of 3–21 % in 95 % percentiles from a student’s *t*-distribution. Robust alternatives to the correlation coefficients in [6] can be computed with a weighting of gradients proportional to the inverse of $abs\left( {\tilde{e}_{i} } \right)$.

### A weighted recovery

In regressions with a positively valued dependent variable (*y*), it is not uncommon to observe an increase in the variance of regression residuals with an increase in *y* [73, ch. 5.1]. A weighted least squares (WLS) approach to model-fitting would be appropriate. If *f* (**x**_*i*_; **b**) was fitted using WLS the recovery of a substitute for ${\text{c}}\hat{\text{o}}{\text{v}}\left( {{\hat{\mathbf{b}}}} \right)$ should also employ a weighting scheme. Equation [8] provides an example.

8 $${\text{c}}\widetilde{\text{o}}{\text{v}}_{wt} \left( {{\hat{\mathbf{b}}}} \right) = \hat{\sigma }_{e}^{2} \left( {{\hat{\mathbf{F}}}^{t} {\mathbf{W}\hat{\mathbf{F}}}} \right)^{ - 1}$$

where **W** is an *n* × *n* diagonal matrix of sum-to-one weights *w*_*1*_, …, *w*_*n*_. In tree biomass models, the weights would typically be proportional to the inverse of, say, DBH_*j*_^2^ which gives the following weights *w*_*j*_ = TDBH^2^ × DBH_*j*_^−2^ where TDBH^2^ is the sum of DBH_*j*_^2^ over the *n* trees. A robust alternative to [8] is obtained by a straightforward extension of [7].

A weighting scheme is also needed when trees for model-fitting were selected by an unequal probability selection scheme. Weights should then be proportional to the inverse of the sample inclusion probability [73, p. 41].

### The number B of resampling replications

The value of *B* was determined adaptively by monitoring the Monte Carlo error as a function of *B* [74]. In our examples we fixed *B* to 800. With this value of *B*, the Monte Carlo error in the determinant of ${\text{c}}\widetilde{\text{o}}{\text{v}}\left( {{\hat{\mathbf{b}}}} \right)$ was less than 4 %.

### Comparing recovered and actual covariance matrices

A recovered substitute for a covariance matrix may vary considerably from an unknown target estimate when the joint distribution of the explanatory variables in the sample used for fitting differs from the joint-distribution in the target population for model application. In our demonstrations we knew, in most cases, the actual estimates of the missing covariance matrix. It is therefore of interest to test the hypothesis of equality between a recovered substitute and the actual estimate. We use Box’s M-test to obtain a Chi square test-statistic and the probability of this test statistic under the null hypothesis of no difference [75, p. 281]. The same test was applied in examples where only the covariance in ${\hat{\mathbf{b}}}$ are unknown.

### Applications

#### Examples from Germany

We demonstrate the above recovery procedures with 15 biomass equations (Table 1) and data (HT, DBH) from 335 plots in the first German national forest inventory (BWI-1987). Note, the data represent trees selected with probability proportional to their basal area. Their mean HT and DBH are therefore larger than the mean of trees selected with equal probability. However, for purpose of a demonstration, this fact is deemed unimportant.

There are four equations (linear, nonlinear, weighted, un-weighted) for each of three species (BEECH, PINE, SPRUCE). Each equation (no. 1–12) were derived from a sample size of *n* = 50 randomly selected trees from five BWI plots. The five plots were excluded from any recovery procedure. In the model fitting, BWI predictions of tree AGB (kg per tree) multiplied with a random uniformly distributed error on the interval [0.9, 1.1] were used as the dependent variable and diameter at a reference height of 1.3 m (DBH) and tree height (HT) were used as predictors. A summary of the BWI data is in Table 7. The remaining three biomass equations (no. 13–15) are generalized species specific AGB equations from Muukkonen and Heiskanen [76]. They are assumed applicable throughout the temperate zone. An analyst may prefer a generalized biomass equation over a local/regional model derived from a relatively small sample size and potentially from a sub-population with a different relationship between AGB and the explanatory variables than in a population targeted for estimation of AGB.

**Table 7:** Means of DBH, HT, and AGB of trees from the 1987 German National Inventory used in this study

	Trees	DBH (cm)	HT (m)	AGB (kg/tree)
BEECH	1,595	35 (15)	25 (7)	1,111 (1,110)
SPRUCE	2,221	30 (12)	24 (7)	492 (460)
PINE	1,221	33 (12)	23 (6)	1,100 (410)

Standard deviations are in parentheses. Note, the mean applies to the population from which 50 trees were selected at random for model-fitting and *B* = 800 sets of 50 trees were selected for the recovery process (a tree used for model fitting was disallowed in the recovery process). See Table 1 for details on sample tree selection.

### Examples from Mexico

Four linear (on a log–log scale) biomass equations [53] with published estimates of $\hat{R}_{adj}^{2}$, $\hat{\sigma }_{e}$, and standard errors of the regression coefficients are used to demonstrate the recovery procedures. The equations (no. 1–4) are in Table 8. Three non-linear biomass equations for *Quercus* spp. [52] with unknown standard errors of the regression coefficients were also included (no. 5–7).

**Table 8:** Above-ground forest tree biomass (AGB kg tree^−1^) equations for five species and a species group in Mexico

Species	#	Equation	$\hat{R}^{2}$	$\hat{\sigma }_{e}^{{}}$ kg	Sample size
*Guazuma ulmifolia*	1	$\log \left( {\hat{\text{AGB}}} \right) = - 1.62 + 2.12\log \left( {\text{DBH}} \right)$	0.97	0.48	18
*Inga vera*	2	$\log \left( {\hat{\text{AGB}}} \right) = - 4.04 + 4.00\log \left( {\text{DBH}} \right) - 0.29\log \left( {\text{DBH}} \right)^{2}$	0.97	0.39	15
*Ochroma pyramidale*	3	$\log \left( {\hat{\text{AGB}}} \right) = - 2.45 + 2.30\log \left( {\text{DBH}} \right)$	0.90	0.96	16
*Trichospernum mexicanum*	4	$\log \left( {\hat{\text{AGB}}} \right) = - 2.82 + 2.42\log \left( {\text{DBH}} \right)$	0.96	0.40	16
*Quercus castenea*	5	$\hat{\text{AGB}} = 0.0416{\text{DBH}}^{2.7154}$	0.97	11.6	38
*Quercus latea*	6	$\hat{\text{AGB}} = 0.0333{\text{DBH}}^{2.6648}$	0.92	12.8	7
*Quercus* spp.	7	$\hat{\text{AGB}} = 0.0342{\text{DBH}}^{2.7590}$	0.93	15.7	45

Equations 1–4 are from Douterlungne D, Herrera-Gorocica AM, Ferguson BG, Siddique I and Soto-Pinto L [53]. Equations 5–7 are from Aguilar et al. [52].

The recovery procedures are demonstrated with data from the 2004–2009 Mexican national forest inventory [47, 48]. Specifically, 132 sample plots and 1,843 trees with known DBH and HT were included (Table 9).

**Table 9:** Summary of tree size (mean DBH cm, mean HT m), stem density of species groups (*N* ha^−1^), and model-dependent predictions of above-ground forest tree biomass (AGB Mg ha^−1^) in the Mexican NFI (2004–2009) plots

Species	State	Stratum	Trees	*n*_*plots*_	DBH	HT	*N* × ha^−1^
*Guazuma ulmifolia*	Chiapas	Mediana subperennifolia	177	12	12.9 (5.1)	5.5 (2.4)	201
*Inga vera*	Chiapas	Alta pernnifolia	37	12	17.1 (9.5)	12.0 (4.1)	103
*Ochroma pyramidale*	Chiapas	Alta pernnifolia	144	20	13.9 (6.2)	10.1 (2.6)	49
*Trichospernum mexicanum*	Chiapas	Alta pernnifolia	612	48	13.9 (6.5)	9.6 (3.3)	156
*Quercus castenea*	Michoacàn	Bosque de encino	416	17	16.6 (7.7)	7.1 (2.6)	612
*Quercus latea*	Michoacàn	Bosque de pino	37	4	17.2 (8.5)	9.5 (3.2)	231
*Quercus* spp.	Michoacàn	Bosque de encino	420	19	16.6 (7.7)	7.0 (2.6)	553

Table entries in parenthesis are standard deviations.

### Application of recovered statistics

The above recovery procedures are motivated by the need to supply inventory estimates of AGB with an estimate of sampling and model errors. The latter is not possible without published or recovered substitutes for missing values of $\hat{\sigma }_{e}$ and ${\text{c}}\widetilde{\text{o}}{\text{v}}\left( {{\hat{\mathbf{b}}}} \right)$.

Under a simple random sampling design, the model error variance in an estimate of a species specific AGB Mg ha^−1^—in a stratum or a population of interest—is obtained by scaling an estimate of the average model error in a tree-level estimates of AGB with an estimate of stem density [40]. The estimator of model error variance per tree in species *s* with a recovered covariance matrix becomes

9 $$\tilde{V}\left( {\hat{{\overline{{\text{AGB}_{s} }} }}} \right) = \frac{{\tilde{\sigma }_{e}^{2} }}{{n_{s} }} + \overline{{\frac{{\partial f\left( {{\mathbf{x}}_{j} |{\mathbf{b}}} \right)}}{{\partial {\mathbf{b}}}}}}^{t}_{{|{\mathbf{b}} = {\hat{\mathbf{b}}}}} {\text{c}}\widetilde{\text{o}}{\text{v}}\left( {{\hat{\mathbf{b}}}} \right)\,\overline{{\frac{{\partial f\left( {{\mathbf{x}}_{j} |{\mathbf{b}}} \right)}}{{\partial {\mathbf{b}}}}}}_{{|{\mathbf{b}} = {\hat{\mathbf{b}}}}}$$

where *n*_s_ is the number of trees in species *s* in the inventory sample, and an over-bar indicates an average over the *n*_*s*_ trees. Let $\hat{\lambda }_{s}$ denote the inventory estimate of the stem density in species *s*. The model error variance in an estimate of AGB Mg ha^−1^ for species *s* is hereafter: $\hat{\lambda }_{s}^{2} \tilde{V}\left( {\hat{{\overline{{\text{AGB}_{s} }} }}} \right) + \left( {\hat{{\overline{{\text{AGB}_{s} }} }}} \right)^{2} \hat{V}\left( {\hat{\lambda }_{s} } \right)$ [70, p. 228], if we assume a zero covariance between stem density and AGB. Under an assumption of independence of model errors across species, the model error variance for a group or all species combined are computed as the sum of the variances of individual species. When a single biomass equation is used for more than one species there will be a covariance of model errors among species sharing a biomass equation [12]. We have restricted results to species specific per tree model errors estimated from [9]. The rationale for bringing these estimates here is that it is easier to gauge whether an estimate of model errors in a tree-level estimate of AGB is reasonable or not. It is much harder to interpret the effects of model errors in the parameters of a biomass equation. Even if a recovered covariance matrix or a recovered residual variance is not on target, the estimated average error obtained via [9] may still be reasonable and a realistic substitute for the error that could otherwise not be estimated.
